# Unnatural Death

**Published:** 1899-04

**Authors:** 


UNNATURAL DEATH.—"Dr. Hill, master of Downing College, Cambridge, read a paper at the recent meeting of the Sanitary Institute of England, with the above title," says *The Times*, London. "He told his hearers that about one million babies were born annually in England; 30,000 of the million would die violent deaths from accident, 30,000 would die unnecessarily from tuberculosis, and 120,000 more from other absolutely preventable causes, such as small-pox, measles, and scarlet fever. Only 45,000 would be allowed to live out their natural lives, and nearly one to twenty would die because the machine was worn out. One-fourth of all the diseases which destroy life are absolutely preventable, and fifteen years would at once be added to its average duration if the practice of hygiene were placed on a level with its theory. Dr. Hill attributed the greater number of the diseases over which the individuals affected by them have personal control to mistakes in eating and drinking."

